# Characteristics and outcome of rapid response team patients ≥75 years old: a prospective observational cohort study

**DOI:** 10.1186/s13049-017-0423-8

**Published:** 2017-08-04

**Authors:** Joonas Tirkkonen, Piritta Setälä, Sanna Hoppu

**Affiliations:** 1 Department of Intensive Care Medicine, Tampere University Hospital, Department of Anaesthesiology and Intensive Care Medicine, Seinäjoki Central Hospital, University of Tampere, PO Box 2000, FI-33521 Tampere, Finland; 2 Emergency Medical Service, FinnHEMS 30, Tampere University Hospital, University of Tampere, PO Box 2000, FI-33521 Tampere, Finland; 3 Department of Intensive Care Medicine, Tampere University Hospital, University of Tampere, PO Box 2000, FI-33521 Tampere, Finland

**Keywords:** Rapid response team, Medical emergency team, Rapid response system, Geriatric, Outcome

## Abstract

**Background:**

Rapid response teams (RRTs) attend severely ill general ward patients whose average 30-day mortality is near 30%. A major part of RRT patients are over 75 years old, but there are no studies on the characteristics and outcome of this geriatric RRT population.

We compared the characteristics and outcome of geriatric RRT sub-population with the RRT patients <75 years old. We further investigated, whether the accumulation of risk factors (RFs) for mortality among the general RRT population predicts a tenuous outcome among the geriatric sub-population.

**Methods:**

Prospective three-year observational cohort study of adult RRT patients in Tampere University Hospital, Finland**.** After identifying independent RFs for 30-day mortality among RRT patients with multivariate logistic regression, we further studied the impact of the accumulation of these RFs among geriatric RRT patients who had no limitations of medical treatment.

**Results:**

A total of 1372 patients were reviewed 1722 times. Geriatric patients (*n* = 449, 33%), when compared to non-geriatric patients, had higher 30-day (33% vs. 21%, respectively; *p* < 0.001) and one-year (54% vs. 35%, respectively; *p* < 0.001) mortality rates. Among the general RRT population, positive RRT criteria as measured by RRT during the review, high comorbidity index, age ≥ 75 years, non-elective hospital admission, medical reason for admission and afferent limb failure were identified as independent RFs for 30-day mortality and classified as feasible to obtain during a routine RRT review. The observed rates of these RFs among the geriatric RRT patients substantially affected their 30-day mortality (e.g. no RFs: 5.3%; one RF: 14%; two RFs: 27%; three RFs: 38%; four RFs: 52%; five RFs: 38%).

**Conclusions:**

One-third of patients reviewed by RRT were ≥75 years old, and age statistics were comparable to previous RRT studies suggesting that this is the case globally. Outcome of geriatric RRT patients is poorer as compared with RRT patients <75 years. However, the outcome is substantially affected by the accruement (or lack) of RFs generally increasing the mortality of RRT patients. Considering these factors during a geriatric RRT review may aid with the decision to either escalate or de-escalate care.

## Background

The rapid response system (RRS) is a pivotal link in the ‘chain of survival’ of an in-hospital cardiac arrest [1]. In cases of in-hospital patient deterioration, rapid response teams (RRTs) form the efferent limb of the RRSs, providing patient assessment, bedside intervention, and rapid escalation of care if deemed appropriate [2]. On the other hand, RRTs initiate limitations of medical treatment (LOMT) in a median of 8% of the reviews, and attend patients with pre-existing LOMT regularly [3, 4].

The average RRT patient is generally around 59–65 years old, presented either as means (± standard deviations) or medians (quartiles) in the RRT literature [4–12]. This suggests that many RRT patients are ≥75 years old. Mortality among the elderly critically ill patients is high, and age is an independent risk factor for worse outcome, although comorbidities, or a lack of them, substantially affect the prognosis [13, 14]. Therefore, high age alone does not equal futility of further treatments no more than young age alone equals better outcome. To assist with RRT reviews in which the initiation of treatment goals, rather than the escalation of care, might be appropriate, Cardona-Morrell and Hillman have developed the CriSTAL tool [15]. The CriSTALs premise is that the RRT patient is ≥65 years old, after which rigorous assessment on patient background is conducted. While the CriSTAL tool seems proficient, statistically in includes on average, half of RRT patients.

There is no data on characteristics and outcome of geriatric (≥ 75 years) RRT patients. It is unknown, whether their prognosis is substantially worse or not as compared with RRT patients <75 years old. Further, it has not been investigated how the accruement of known risk factors for mortality among the general RRT population, or lack of them, influence on the prognosis of geriatric RRT patients.

## Methods

### Aim and design

We aimed to compare the characteristics and outcomes of RRT patients ≥75 years old with younger adult RRT patients in a three-year prospective observational single centre cohort study. We further investigated the independent risk factors for fixed 30-day mortality among the whole study cohort and then tested the hypothesis, that the accruement of these risk factors affect the outcome of geriatric RRT patients without preceding LOMT.

### Ethics

The Ethics Committee of the Tampere University Hospital (Tays) approved the study protocol (Approval no: R10111). Patient consent was waived as no interventions were conducted.

### Hospital and RRT

Tays is one of five tertiary referral centres in Finland, with 71,000 somatic admissions annually. It has a closed model, mixed surgical-medical intensive care unit (ICU) with 24 beds and approximately 2100 admissions per year.

Tays has a RRS that includes regular training of the wards’ RRS-responsible nurses, who, in turn, distribute this knowledge to their homeward colleagues. The wards use dichotomised RRT activation criteria (heart rate < 40/min or >140/min, systolic blood pressure < 90 mmHg, peripheral arteriolar oxygen saturation < 90%, respiratory rate < 5/min or >24/min and decrease in state of consciousness), in addition to the subjective ‘nurse worried’ criterion (no objective criteria are required in case a nurse is worried that a patient is deteriorating). The RRT comprises an ICU physician (team leader) and two ICU nurses. The RRT has a two-tier approach when triggered; if the review is not assessed as immediately life-threatening, the two RRT nurses may first attend the patient before reporting to the RRT leader.

### Definitions

The term ‘RRT patient’ refers to a hospitalised individual requiring one or several RRT attendances. Regular follow-up visits are sometimes issued for discharged ICU patients; in this study, these scheduled ‘outreach visits’ were not considered RRT activations. The age limit for a patient to be defined as ‘geriatric’ or ‘aged’ varies substantially in the intensive care and resuscitation literature (≥ 60–80 years) [13–19]. The European Union Geriatric Medicine Society refers to patients >60 as aged [20]. Defining patients 60–65 years old as ‘geriatric’ did not seem appropriate in this study, considering the age distribution of the general RRT cohorts [4–12] and mere the fact that the statutory flexible pension age in Finland is 63–68 years. Therefore, we defined RRT patients ≥75 years old as ‘geriatric’ RRT patients in this study.

### Data collection

Between 1 May 2012 and 30 April 2015, we collected data prospectively on RRT activations per the Utstein Style. This collection was part of an ongoing project of RRT data gathering (the third component of the RRSs, Jones et al. 2011) [2, 21]. Specific patient data were obtained from patient records, data on afferent limb failure (documented positive MET activation criteria 20–360 min before the RRT activation) were obtained from electronic nursing records, and long-term mortality data were retrieved from the Finnish Population Register Centre [22].

### Exclusion criteria

RRT activations for paediatric patients (< 18 years) and hospital visitors (outpatients) were excluded. RRT activations to the ICU, operation rooms and emergency department were also excluded. Finally, RRT reviews involving cardiac arrests and repeat RRT reviews were excluded.

### Statistical analysis

Data are presented as numbers (percentages) unless otherwise indicated. Chi-square and Mann–Whitney *U*-tests were used for comparisons between groups. Multivariate logistic regression was applied with the ‘ENTER’ method to investigate those factors independently associated with worse outcomes within the entire RRT population. Age was inputted as a dichotomised variable to reflect the study’s hypothesis. A Hosmer-Lemeshow test was conducted to present the goodness-of-fit of the model. The identified risk factors that would be feasible to obtain during routine RRT review were then tested as dichotomized variables among the no-LOMT geriatric sub-population.

Tests were two-sided; *p* < 0.05 was considered significant and 95% confidence intervals were reported where appropriate. SPSS version 20 for Windows (SPSS Inc., Chicago, IL, USA) was used.

## Results

### Study cohort

**Fig. 1:**
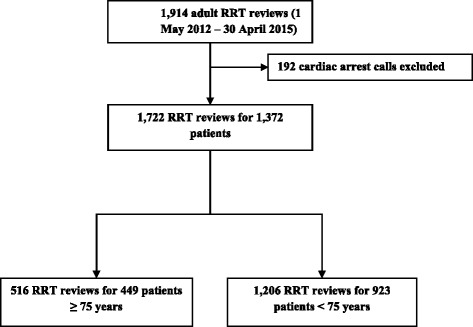
Study cohort. RRT, rapid response team

**Fig. 2:**
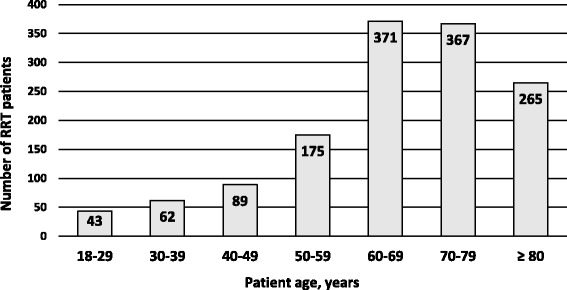
Distribution of the 1372 RRT patients according to age. RRT, rapid response team

During the study period, 1914 RRT reviews meeting the inclusion criteria were observed. In 192 of these, the patient suffered a cardiac arrest; these reviews were excluded from further analysis. The final cohort comprised 1372 RRT patients who were attended a total of 1722 times (Fig. 1). Figure 2 presents the age distribution of the cohort: 1003 (73%) RRT patients were ≥60 years old, and 632 (48%) ≥ 70 years old. The median age of the RRT patients was 69 (59, 78), and the mean age was 67 ± 16 (both skewness and kurtosis between −1.0 and +1.0).

### Geriatric RRT patients vs. non-geriatric RRT patients

Every third RRT patient was considered geriatric patient (449, 33%) (Fig. 1). Geriatric patients were more likely to be female, to be admitted as surgical patients and to present comorbidity burden than patients <75 years old (Table 1). RRT review characteristics were otherwise relatively comparable, though geriatric patients were more likely to receive a new LOMT and were seldom admitted to intensive care. Among the patients admitted to intensive care, survival rates and length of stay were equal for geriatric and non-geriatric patients.

**Table 1:** Characteristics of RRT patients and their first RRT reviews, ≥ 75 years vs. < 75 years old

	≥ 75 years (*n* = 449)	< 75 years (*n* = 923)	*p-value*
Patient characteristics			
Age (median; Q_1_, Q_3_)	82 (78, 85)	63 (52, 69)	< 0.001
Sex (male)	240 (54)	581 (63)	0.001
Medical patient	155 (35)	405 (44)	0.001
CCI (median; Q_1_, Q_3_)	2.0 (1.0, 4.0)	2.0 (0.0, 3.0)	< 0.001
Coronary artery disease	108 (24)	102 (11)	< 0.001
Chronic heart failure	124 (28)	99 (11)	< 0.001
Peripheral artery disease	55 (12)	83 (9.0)	0.063
Cerebrovascular disease	86 (19)	108 (12)	< 0.001
Diabetes	112 (25)	218 (24)	0.620
Chronic obstructive pulmonary disease	55 (12)	100 (11)	0.449
Renal insufficiency	48 (11)	77 (8.4)	0.162
Malignancy	117 (26)	227 (25)	0.579
Elective hospital admission	345 (77)	698 (76)	0.621
Length of hospital admission (days, median; Q_1_, Q_3_)	8 (4, 14)	11 (6, 23)	< 0.001
Preceding ICU admission	52 (12)	198 (22)	< 0.001
Preceding LOMT	38 (8.5)	48 (5.2)	0.019
Surgery 0–24 h before the review	73 (16)	107 (12)	0.016
RRT review characteristics			
Days in hospital before the review (median; Q_1_, Q_3_)	2 (1, 5)	2 (1, 7)	0.001
Review during on-call time^a^	340 (76)	700 (76)	0.963
Afferent limb failure^b^	128 (37)	273 (36)	0.650
Length of RRT review (min) (median; Q_1_, Q_3_)	27 (20, 39)	30 (20, 41)	0.044
Reason for RRT activation			
• Respiratory	156 (45)	304 (40)	
• Circulatory	69 (20)	142 (19)	
• Neurologic	47 (14)	128 (17)	0.297
• Multiple	14 (4.1)	38 (5.0)	
• Other^c^	59 (17)	153 (20)	
Vitals documented by RRT			
• AVPU ≤3 or GCS ≤ 13	129 (29)	233 (25)	0.176
• Heart rate < 40 or >140 /min	42 (9.4)	75 (8.1)	0.452
• Systolic blood pressure < 90 mmHg	61 (13)	126 (14)	0.962
• Respiratory rate < 5 or >24 /min	175 (39)	351 (38)	0.757
• SpO_2_ < 90%	139 (31)	238 (26)	0.047
• None of the above	118 (26)	246 (27)	0.866
RRT intervention			
• Fluids	165 (37)	305 (33)	0.175
• Oxygen			
o Intubation	20 (4.5)	48 (5.2)	
o CPAP	42 (9.4)	105 (11)	0.300
o Mask	226 (50)	417 (45)	
• Medications	100 (29)	239 (31)	0.450
New LOMT	57 (13)	43 (4.7)	< 0.001
Transfer to ICU	69 (15)	265 (29)	< 0.001
• ICU LOS (days, median; Q_1_, Q_3_)	2 (1, 3)	3 (1, 5)	0.053
• Died in intensive care	9/69 (13)	31/265 (12)	0.759

Data are presented as numbers (percentages) if not otherwise indicated. *RRT* rapid response team, *CCI* Charlson comorbidity index; Malignancy, malignant solid tumor or hematologic malignancy; *ICU* intensive care unit, *LOMT* limitations of medical treatment, *AVPU* alert, voice, pain, unresponsive, *GCS* Glasgow coma scale, *CPAP* continuous positive airway pressure, *LOS* length of stay

^a^On-call time: Other than Monday − Friday 8.00 a.m. to 3.00 p.m.

^b^Documented positive MET activation criteria 20–360 min before the RRT activation

^c^Includes the calls triggered by ‘staff worried’ criterion

Table 2 presents the outcomes of the study population. Geriatric patients had higher 24 h and hospital mortality, were rarely discharged home and had higher fixed mortality for up to one year when compared to non-geriatric patients.

**Table 2:** Outcome of RRT patients ≥75 years vs. < 75 years old

Patient outcome	≥ 75 years (*n* = 449)	< 75 years (*n* = 923)	*p*-value
New RRT review	49 (11)	177 (19)	< 0.001
24 h mortality	37 (8.2)	46 (5.0)	0.018
Hospital mortality	104 (23)	158 (17)	0.008
Discharged alive to			
• Home^a^	52 (15)	296 (39)	
• Other hospital^b^	175 (51)	302 (40)	< 0.001
• Primary care ward	118 (34)	167 (39)	
30-day mortality	148 (33)	193 (21)	< 0.001
180-day mortality	206 (46)	281 (31)	< 0.001
One year mortality	242 (54)	323 (35)	< 0.001

Data are presented as numbers (percentages) if not otherwise indicated. *RRT* rapid response team

^a^Includes discharge to nursing home if this was patient’s residence before the hospital admission

^b^Includes local district hospitals

### Independent risk factors for 30-day mortality among the study cohort

Table 3 presents the results of the multivariate regression analysis of the factors independently associated with 30-day mortality among the 1322 RRT patients. Preceding LOMT, positive RRT criteria as measured by RRT during the review, high comorbidity index, age ≥ 75 years, non-elective hospital admission, medical reason for admission and afferent limb failure were identified as independent risk factors.

**Table 3:** Multivariate logistic regression analysis of factors independently associated with 30-day mortality, whole cohort (*n* = 1372)

Multivariate analysis			
	Odds ratio	95% CI	*p*-value
Preceding LOMT	3.84	2.38–6.20	< 0.001
Positive RRT criteria measured by RRT	2.24	1.58–3.16	< 0.001
CCI	1.18	1.11–1.26	< 0.001
Age > 75 years	1.75	1.33–2.30	< 0.001
Non-elective hospital admission	1.99	1.40–2.83	< 0.001
Preceding ICU admission	0.64	0.44–0.95	0.025
Medical patient	1.37	1.04–1.80	0.026
Afferent limb failure	1.31	1.00–1.71	0.049
Surgery within 24 h	0.74	0.48–1.15	0.180
Sex (male)	1.16	0.88–1.52	0.278

The Hosmer-Lemeshow goodness-of-fit Chi-square (8) with *p* = 0.335 indicated a good fit of the model. *RRT* rapid response team, *LOMT* limitations of medical treatment, *CI* confidence interval, *CCI* Charlson comorbidity index, *ICU* intensive care unit

### Effect of the accumulation of the risk factors on the outcome of geriatric RRT patients without preceding treatment limitations

**Fig. 3:**
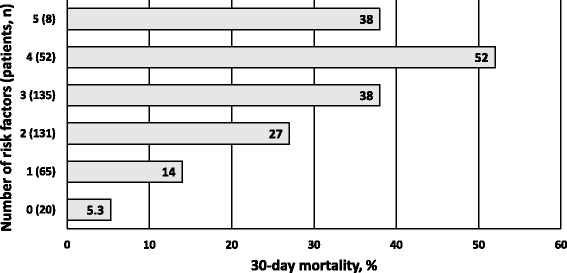
The cumulative impact of risk factors found in multivariate regression model on 30-day mortality of RRT patients ≥75 years old and without treatment limitations (*n* = 411). Risk factors were identified as positive RRT criteria measured by RRT, Charlson comorbidity score ≥ 5, non-elective hospital admission, medical reason for admission, and afferent limb failure. RRT, rapid response team

The above-mentioned risk factors were tested in the geriatric sub population without preceding LOMT (*n* = 411). By definition, the factors of age ≥ 75 years and preceding LOMT were not applied, and the Charlson comorbidity score was dichotomised to <5 and ≥5 (the latter indicating severe comorbidity). Figure 3 presents the impact of the accruement of these risk factors (or lack of them). Geriatric patients with none of these factors had a fixed 30-day mortality of just 5.3%, whereas patients with four of these factors had a 52% 30-day mortality (*p* < 0.001). Only eight geriatric patients had all five risk factors.

## Discussion

### Key findings

This prospective observational cohort study revealed that every third RRT patient is ≥75 years old. Since the median (and mean) age of the cohort was comparable to other studies around the world, it seems that RRTs globally are faced with the same challenges related to ageing populations. As may be expected, the geriatric sub population was, in general, more prone to worse outcomes. However, advanced age alone does not per se equal futility of advanced treatments, though it is an independent risk factor for 30-day mortality. Indeed, the possible futility and ethics of escalating geriatric RRT patients’ care should be carefully weighed when these patients present multiple known risk factors for 30-day mortality among the general RRT population. On the other hand, lack of these risk factors during a geriatric RRT review indicate that geriatric patient’s prognosis may be substantially better than RRT patients’ prognosis in general.

### Age of RRT patients

RRT studies from Australia, New Zealand, the United States of America and Sweden report the median or mean age of RRT patients to range between 66 and 74, and these values are in line with our findings [4, 5, 7–9, 11, 12]. One study from Brazil reported a lower mean age of 63 years, while one study from the United Kingdom reported a higher median age of 76 years indicating that some variability exists [6, 10]. Nevertheless, it may be concluded that, in general, a major part of RRT patients are of truly advanced age (≥ 75 years). Therefore, our findings can be generalised to the challenges related to ageing RRT patients around the world. Furthermore, the percentage of the RRT patient population that is geriatric can only be expected to increase with the ageing populations in Western countries; this has already been observed among ICU patients [19].

### Comparison of geriatric RRT patients with younger RRT patients

The comorbidity burden was not substantially higher among the geriatric RRT patients, although the difference was statistically significant. The results suggest that younger RRT patients often have moderate to severe comorbidies as well. RRT trigger reasons were comparable, and vital signs were documented as often abnormal in both sub cohorts. Perhaps the most important finding was, that the short-term outcomes of patients admitted to ICU did not differ between the geriatric and younger RRT patients. Thus, the RRT seemed to conduct well-founded patient selection despite often operating during on-call hours, during which the support from the parent unit is limited. Long-term fixed mortality rates were higher in geriatric sub cohort, but it should be acknowledged that mortality rates among younger RRT patients were high as well.

### Risk factors for 30-day mortality

The previously identified association of several independent risk factors with hospital mortality or 30-day mortality was confirmed for 30-day mortality in this study [6, 7]. In addition, the cumulative impact of basic diseases was inputted as continuous variable, the Charlson comorbidity index score, to the multivariate logistic regression model, and it was shown to be independently associated with worse outcome [23]. Finally, as a major part of the RRT calls are triggered for non-physiological reasons (e.g. the ‘nurse worried’ criterion), we also included whether the patient fulfilled the RRT activation criteria during the review as a variable in the model. Positive activation criteria recorded by the RRT were also associated with worse outcomes, as could be expected.

### Identified risk factors as outcome predictors for geriatric patients

Measured at any given point, from 24 h to one year after the visit, geriatric patients had higher mortality rates. This, however, did not reveal anything about the prognosis of an individual geriatric patient. We found that the prognosis of geriatric patients varied substantially, depending on the accruement of risk factors known to be associated with poor prognosis among RRT patients. In fact, the geriatric RRT patients with zero or one risk factor had lower 30-day mortality rate than the RRT patients <75 years old despite their median age was just 63 years. Worth discussing is also that afferent limb failure, also known as delayed RRT activation, was included as one of the five factors tested here. While this factor represents a clear system failure, unfortunately patient’s prognosis is indisputably worse if his/her condition has been allowed to deteriorate for hours without interventions. In some cases ICU physicians are forced to evaluate, whether a patient has already deteriorated beyond salvation.

All five risk factors are feasibly and quickly obtained from the ward nurses or recent patient records during a RRT review. Therefore, these factors could also be taken into consideration during on-call hours, which have limited resources and time but comprise over three thirds of RRT activations. In fact, the CriSTAL investigators themselves found in their recent retrospective case-control analysis that several factors normally considered important when initiating LOMT discussions (disability, previous hospital admissions, proteinuria, etc.) were not available even in a study setting, where the lack of time for thorough review of patient records is not a factor [16]. Perhaps adding up the known risk factors for worse outcome in our study could also assist the RRT physician when a RRT review with ethical considerations regarding a geriatric patient’s best interests is urgently needed. On the other hand, lack of these risk factors should suggest that escalating care for a deteriorating geriatric RRT patient is not futile at all.

### Limitations

This study is of prospective design, but it was conducted in a single centre in a Nordic university hospital. While we suggest that the major part of RRT activations concern geriatric patients across all continents, cultural differences in clinical practices and RRT usage, including LOMT, may vary substantially. In fact, the whole RRS concept varies substantially between centres and countries [4–12]. The methodology of identifying risk factors from a large RRT population and then testing them in a small sub-population derived from the same primary cohort has several limitations, so our results should only be considered preliminary. Moreover, results of a multivariate regression model can never dictate therapies, merely provide suggestions for clinicians. However, the identified risk factors were comparable to those previously reported and probably apply in other institutions too.

## Conclusions

Every third RRT patient was ≥75 years old in this study, and in the light of previous studies, this seems to be the case globally. Geriatric RRT patients have poorer short- and long-term outcomes when compared to RRT patients <75 years old and age is an independent risk factor for mortality. However, the cumulative impact of the factors independently associated with worse outcomes among the general RRT population substantially influences outcomes among geriatric RRT patients. Geriatric patients with none of these risk factors had substantially better outcome than RRT patients in general.

## Abbreviations

CriSTAL: Criteria for Screening and Triaging to Appropriate aLternative care
ICU: Intensive care unit
LOMT: Limitations of medical treatment
RF: Risk factor
RRS: Rapid response system
RRT: Rapid response team
Tays: Tampere University Hospital
